# New York Academy of Medicine.—Section on Orthopedic Surgery

**Published:** 1891-08

**Authors:** 


### Society Proceedings.

### NEW YORK ACADEMY OF MEDICINE.—SECTION ON ORTHOPEDIC SURGERY.

*Stated Meeting, May 15, 1891.*

SAMUEL KETCH, M. D., Chairman.

Dr. T. HALSTED MYERS presented a case of marked rickets, called congenital on the mother's positive assertion that the greatly enlarged epiphyses of the tibia, femora, and radii were present when she first examined the child a few days after birth. The sternum at that time, she noted, also was abnormally prominent. She had been in very good health all through gestation, and the father was a healthy man. No specific history could be obtained.

At present the child is six years of age, and presents all the deformities of rickets in a marked degree, except that the head is well shaped, and there is a marked increase of the normal dorsal curve of the spine, rather than the dorso-lumbar kyphosis usually found in these cases. An unusual degree of permanent knee and hip flexion also exists, and the patient assumes, when resting, the hand-to-knee position of Pott's disease. The epiphyseal tenderness present seemed to indicate an active stage of the disease. After being nursed nine months, the child had a mixed diet, not especially starchy, nor lacking in animal fats.

THE CHAIRMAN thought it not improbable that the spinal symptoms were the result of an acute lesion occurring coincidently with this diathesis. In cases of simple kyphosis which he had examined, one of the points in the differential diagnosis had been the absence of psoas contraction, and in most of these cases the curve, unlike this one, disappeared when the patient was in the prone position.

Dr. JOHN RIDLON exhibited the photograph of a patient, nineteen years old, who had had exactly this position all his life. There were additional curves in both the tibia and femora, which had developed gradually during his growth. It was worthy of note that psoas contraction was also present in his case.

Dr. NEWTON M. SHAFFER had seen psoas contraction in these cases of rachitis. The case just presented was not, in his opinion, one of tubercular disease of the spine, but a sensitive condition of the cancellous structure in the bodies of the vertebræ which simulates Pott's disease. He had never seen a case which he could consider one of congenital rachitis, and he was inclined to look upon this one as an instance of rachitis acquired at a very early age. It was not uncommon to find in rachitic patients a condition of the muscles somewhat resembling that found in tubercular joints. He was reminded of a case which he had seen in St. Luke's Hospital, in which there was a very sensitive joint, associated with muscular symptoms which might suggest hip-joint disease, but these were simply due to hyperemia of the epiphysis occurring in a rachitic subject, and in due time, with proper attention to nutrition, these symptoms disappeared.

Dr. Ridlon said that he had expected to present a patient illustrating certain peculiar conditions found in persons who had the caisson disease. His patient had been working in compressed air for sixteen years, and during the past year had had forty or more attacks of the cramps which are peculiar to this disease. Associated with these were stiffness, gradual shortening, and outward rotation of the right lower extremity, with a direct upward dislocation of the hip for a distance of three-quarters of an inch. This man had informed him that he knew of a number of others who had been working in compressed air, who had paralysis with shortening of the limb.

The Chairman said that he had seen a man, forty years of age, who had been a caisson worker and diver, and who presented an affection of both hips. There was very little motion except in abduction. There was no history of rheumatism, or other constitutional disorder.

Dr. Shaffer had recently seen, at the Orthopedic Dispensary, a caisson worker, who presented bilateral hip symptoms, and who was scarcely able to walk. In this case, the symptoms were those of a pronounced rheumatic type, and the changes were apparently due to rheumatic arthritis.

Dr. Ridlon also exhibited photographs of the latest modification of Grattan's osteoclast, and of some of the cases which this surgeon had treated by means of the instrument. He now used it for forcibly correcting club-feet, and in the opinion of the speaker it was the handiest and most efficient contrivance of its class that he had seen.

### NON-UNION AFTER OSTEOTOMY IN A CASE OF SEVERE RACHITIS.

THE CHAIRMAN presented a little girl with a very exaggerated form of rickets, whose symptoms indicated that the disease was still active. The chief point of interest was the fact that, about three years before, a skilful surgeon had performed osteotomy upon her for the correction of a severe form of bow-legs, and this had resulted in non-union. This case showed the folly of operating in the presence of such a virulent form of rachitis. The treatment, in his hands, had consisted in the application of a co-aptation splint, and of a perineal crutch, which, by means of a snap-joint, allowed motion at the knee, but prevented dangerous traumatism, and favored locomotion. The idea of the apparatus was to favor locomotion rather than to attempt to secure union.

Dr. ROYAL WHITMAN doubted if this treatment would lead to union of the fragments, for the end of the bones, in such cases, become extremely hard, and usually require to be removed before union can be secured.

Dr. R. H. SAYRE thought the non-union, in this case, might have resulted from the fact that the deformity was so great that, in order to correct it, a considerable interval must have been left between the ends of the bone after the osteotomy.

### WHEN SHALL WE DISCONTINUE MECHANICAL TREATMENT IN HIP-JOINT DISEASE? WITH REMARKS ON THE SYMPTOMS AND TREATMENT.

The paper of the evening, bearing the above title, was read by Dr. NEWTON M. SHAFFER.

The writer called attention to the difficulty which often existed in deciding this question, and entered a strong protest against the use of an anesthetic as an aid in reaching a conclusion. Ether, it was claimed, would remove the reflex muscular protection of the joint in osteitic disease, and, with nature's protection removed, undue traumatism might be inflicted, and under the influence of this traumatism, encysted tubercular material might be broken up and a fresh infection occur. He recognized the fact that tubercular disease must run a long course, and he had long since ceased to expect any "short cut" in the treatment of these conditions. Scientific mechanical treatment places the joint under the best local conditions for repair, and aids nature, by climatic and other influences, in reaching the period of self-limitation; but, after disintegration of the joint had once occurred, there was no apparatus that would cure hip disease, any more than a splint would cure a fractured thigh. Reference was made to the report by Dr. Lovett and the author on The Ultimate Results of the Mechanical Treatment in Hip-Joint Disease, published in 1887. Notwithstanding the great care exercised, and the four years' limit which governed the investigation of the cases reported upon, there had been several relapses.

Attention was then called to the fact that many surgeons ignore the neuro-muscular symptoms of hip-joint disease, and to the fact that anesthesia removes the true reflex muscular spasm; that the absence of pain was not a safe criterion; that the absence of abscess afforded no positive evidence of the cessation of the disease; and that the patient could stand a very severe concussion of the joint without pain or flinching, and yet be suffering from extensive and progressive tubercular disease; that abscesses and sinuses might exist (unconnected with the joint), and yet the patient be free from the necessity of mechanical treatment; and that sinuses might close, and abscesses disappear, with active disease present.

The author then stated that only two elements existed upon which a positive opinion could be based, viz.: (1) the gait and attitude of the patient, and (2) the character of the resistance to joint motion thus obtained. He divided the limp into three classes: (1) the limp of true disease; (2) the limp of a vulnerable joint in the convalescent stage, and (3) the limp of shortening and disease, all of which were described.

The important element, however, was the neuro-muscular protection of the articulation. He described it as a purely involuntary and instinctive effort on the part of nature to prevent traumatism. Without this element present, we are unable, as a rule, to make a diagnosis of hip disease, and if it were not present there would be no deformity. The mechanical treatment should be directed not only to the deformity, but to the disease; and the necessity of controlling the knee was pointed out. The author's experience led him to advise the use of the old Taylor traction splint, with the rigid pelvic band, and double perineal pad, in securing the proper modification of traumatism at the hip, and in controlling the knee; and he spoke rather disparagingly of any splint in the state of convalescence which permitted motion at the knee. He also stated that we need not fear the effect of prolonged mechanical treatment as much as the unheeded cry of the diseased joint for proper protection.

The following conditions contra-indicated the removal of the apparatus; if manual concussion produces pain or flinching; if there is considerable deformity without anchylosis; if there is a true joint limp, or if there are abscesses or sinuses connected with the joint; or if there is a true reflex; muscular spasm limiting movement slightly in all directions; if there is almost perfect flexion, with the other movements considerbly or markedly limited; if flexion and abduction and adduction are excellent, with rotation and extension limited; and, finally, if all the movements are nearly normal, except rotation inward during flexion (the limitations being due to the neuro-muscular protection), it is not safe to discontinue mechanical protection. Rotation inward during flexion is always the last motion to recover, and this may remain for several years after all the other signs have disappeared, and, in many cases, it still remains after the joint had recovered; but, in the latter case, its reflex character disappears.

Attention was called to the fact that, even after the limp had entirely disappeared, a relapse may occur. A recent case, occurring at St. Luke's Hospital, was cited as an example. From this and other similar cases, the author draws the conclusion that there is a recognizable stage of hip-joint disease which ante-dates the limping stage.

Excising the joint was then referred to, and the conclusion reached that in the absence of signs and symptoms by which we can exactly determine the extent of the lesion, and with the great difficulty, not to say impossibility, of a complete excision of the acetabular portion of the joint, excision of the joint was an unsatisfactory and, in many cases, an unsafe operation, and that mechanical treatment, while more difficult, and requiring special training to make it successful, promised more satisfactory results, both as to life and usefulness of the affected member.

The conclusions were as follows:

In the first apparent stage of tubercular disease of the hip-joint, when there is no deformity present, and where we have only the neuro-muscular signs or the slight limp, or both, to guide us, as well as in the more severe forms of the disease, where tubercular disintegration of the joint had commenced, and when the muscular protection of the articulation is more pronounced, the only safe guides for discontinuing mechanical treatment are (1) the absence of the expressive attitude and gait of tubercular osteitis of the hip-joint, and (2) an essential modification or an abolition of the instinctive neuro-muscular protection of the articulation ; (3) that in all but exceptional cases a relapse as to the deformity, or the disease, or both, is likely to occur as the result of the traumatism of locomotion, unless proper mechanical protection is maintained until the articulation is free from true reflex muscular spasm, or is anchylosed.

DISCUSSION.

Dr. A. B. JUDSON shared in the general wish for more certain indications in the convalescent period. He agreed with Dr. Shaffer in thinking that the reflex or neuro-muscular signs are by far the most valuable indications of the condition of the joint. He never resorted to the use of ether in examining the joint, or to the more atrocious barbarism of striking the patient's heel till pain is produced. A patient of his had described the sensation of reflex action by saying that it resembles the general sensation felt in a swing when the descent from the highest point begins.

As but few of the superficial muscles are found by palpation to be contracted, he thought it likely that the intrinsic muscles, those beyond the reach of palpation, are chiefly affected, and suggested that, probably, the muscles exhibiting those phenomena are those which, like the adductors, have their origin and insertion in the bones which enter directly into the composition of the joint. The patient or the mother is sometimes alarmed by the discovery of the rigid adductor muscle, which is thought to be a morbid growth, or an abnormal bone, till it is shown that a similar thing is produced on the well side when an effiort is made which throws the adductors of that side into tonic contraction.

He thought it well to note the variety of these reflexes. Fixation of the joint is produced by a tonic contraction, but motion, especially in the early and convalescing periods, is asserted at a varying point when a considerable arc has been traversed, by a muscular spasm often recognized by the patient. Dr. Fayette Taylor, observing with still greater refinement, had classed "reluctance to relax," shown by the circumarticular muscles, among the reflex signs of incipient osteitis.

Dr. R. H. SAYRE said that if the signs of reflex spasm continued, there was but little doubt that an unprotected joint would become deformed. An experimental removal of the apparatus seemed to be the only way of deciding about discontinuing mechanical treatment. It was true that the late Mr. Thomas said that anyone who could not tell the day and hour when the disease stopped, ought not to treat joint diseases; but his remarkable insight would appear to be quite exceptional. The existence of internal rotation and flexion he did not consider to be so significant as the author stated, for a hip which recovered with impaired motion was not necessarily a vulnerable one. It was highly important to distinguish carefully between the limitation of motion resulting from a deposit around the joint, and that due to reflex spasm. In the former, there was not likely to be any damage to the joint from the removal of protecting apparatus.

Dr. WHITMAN thought the case cited in the paper, which proved fatal as a result of prolonged suppuration, should have been treated by excision, for he had seen a number of apparently hopeless cases of this kind recover after such an operation.

Dr. H. W. BERG thought that reflex muscular spasm was an unconscious as well as a conservative effort of nature, and, therefore, he could not understand how a description could be given by Dr. Judson's patient of the sensation produced by this spasm.

Dr. JUDSON replied that the reflex action in question, when spasmodic, resembles the ordinary reflexes, such as respiration and nictitation, in being recognized by the patient.

Dr. MYERS said that the case at St. Luke's Hospital, referred to, had been examined repeatedly for six weeks after all pain, deformity, and limp had disappeared, and the reflex muscular spasm was always detected. He had found the suggestion of Dr. Shaffer to carefully avoid outward rotation during flexion tests, a very practical and valuable one, and had also noted that the same care should be used in testing abduction to avoid outward rotation, as the reflex muscular spasm at times could only be detected at the very extremes of motion. Dr. Myers said that during his observation of hip joint disease under the tuberculin treatment at St. Luke's Hospital, he had made daily careful examination, and had come to the conclusion that the reflex muscular spasm was the first symptom affected. In the more marked cases, the symptoms, though lasting but a few days, exactly resembled the usual exacerbation of the disease, with increase of reflex spasm, less motion, or even deformity, increase of pain and sensitiveness, and recurrence of night cries. In less marked reactions, several times the reflex muscular spasm become more alert, though there was no rise of temperature, nor appreciable increase of joint sensitiveness, or decrease of motion. He believed with the reader of the paper, that this spasm was the first and the last symptom in hip-joint disease. The tubercular process, he thought, was self-limited, and, therefore, the indication to avoid traumatic reinfection was imperative.

Dr. Samuel Lloyd referred to a case of hip-joint disease, which he had had under observation, in which there was a recurrence after a period of nearly nine years. The proper time for the removal of apparatus could only be determined by experiment in each case. Lately he had been endeavoring to assist the mechanical treatment of suppurative cases by injecting a ten per cent. emulsion of iodoform in glycerine, and the results so far had been quite beneficial.

The Chairman thought that the question of the self-limitation of tubercular disease would account very satisfactorily for the varying results obtained in the removal of apparatus. The only absolutely reliable guide was the existence of reflex muscular spasm, and although he had studied this symptom carefully for many years, he was compelled to admit that in a certain proportion of cases, it was very easily confounded with the mechanical resistance resulting from changes about the joint. The cases of so-called relapse he was inclined to consider as a development of new foci of the disease.

He had been interested in the author's remarks about the fallaciousness of the ether test, and the useless traumatism often inflicted upon joints by improper manipulation and examination.

Dr. Shaffer, in closing the discussion, said with reference to the sensations of the patient resulting from reflex spasm, that as long ago as 1876, a very intelligent gentleman had compared this sensation to that experienced upon attempting to dodge a blow aimed at the stomach. The intrinsic muscular element would not explain the phenomena of reflex spasm, as was shown in knee-joint disease, where the gastrocnemius muscle resists attempts at moving the knee-joint, but allows of motion at the ankle-joint. He believed that reflex spasm required for its development a peculiar specific irritation within the joint, probably of the nerves in the epiphysis. The very fact that this spasm is beyond the control of the patient's will, renders it such a reliable guide in diagnosis and in deciding when to remove the apparatus. As regards the question of excision in the case referred to, he had not presented the full history of the case, and consequently had omitted to say that the father absolutely refused to give his consent to this operation. He thought all orthopedic surgeons recognized the self-limitation of tubercular disease, especially since the able paper published some years ago by Dr. Austin Flint. With regard to relapses, he felt that the traumatism of locomotion was sufficient in many cases to destroy the encysted condition of the tuberculous deposit about the joint, and hence, to produce a fresh infection with tubercular material of the vulnerable tissues in the capsule.

### IRON CASTS AND CO-APTATION SPLINTS.

Dr. Whitman spoke of the advantage of employing iron splints in cases, particularly about the feet, where perfect apposition is desirable. A rough cast of the part is taken in plaster-of-Paris, and sent to the iron founder, who produces an iron cast at an average cost of one dollar. On this cast very light metal splints can be readily and accurately molded.

